# Worth the ‘EEfRT’? The Effort Expenditure for Rewards Task as an Objective Measure of Motivation and Anhedonia

**DOI:** 10.1371/journal.pone.0006598

**Published:** 2009-08-12

**Authors:** Michael T. Treadway, Joshua W. Buckholtz, Ashley N. Schwartzman, Warren E. Lambert, David H. Zald

**Affiliations:** 1 Department of Psychology, Vanderbilt University, Nashville, Tennessee, United States of America; 2 Neuroscience Graduate Program, Vanderbilt University, Nashville, Tennessee, United States of America; 3 Vanderbilt Kennedy Center, Nashville, Tennessee, United States of America; 4 Department of Psychiatry, Vanderbilt University Medical Center, Nashville, Tennessee, United States of America; University of Granada, Spain

## Abstract

**Background:**

Of the putative psychopathological endophenotypes in major depressive disorder (MDD), the anhedonic subtype is particularly well supported. Anhedonia is generally assumed to reflect aberrant motivation and reward responsivity. However, research has been limited by a lack of objective measures of reward motivation. We present the Effort-Expenditure for Rewards Task (EEfRT or “effort”), a novel behavioral paradigm as a means of exploring effort-based decision-making in humans. Using the EEfRT, we test the hypothesis that effort-based decision-making is related to trait anhedonia.

**Methods/Results:**

61 undergraduate students participated in the experiment. Subjects completed self-report measures of mood and trait anhedonia, and completed the EEfRT. Across multiple analyses, we found a significant inverse relationship between anhedonia and willingness to expend effort for rewards.

**Conclusions:**

These findings suggest that anhedonia is specifically associated with decreased motivation for rewards, and provide initial validation for the EEfRT as a laboratory-based behavioral measure of reward motivation and effort-based decision-making in humans.

## Introduction

Major depressive disorder (MDD) is a heterogeneous and etiologically complex disorder. When using group designs, this variability can impede progress by masking important differences across MDD subtypes, as the diagnosis of MDD relies on clinical presentation rather than a pathophysiologically-based nosology [1]. One method of addressing this challenge is the identification of psychopathological endophenotypes for psychiatric disorders, which can be used to identify specific mechanisms that may mediate the relationship between biological and environmental diatheses and clinical phenotypes[2], [3].

To date, one of the most promising psychopathological endophenotypes in MDD is anhedonia [3]. Anhedonia— described as a decreased motivation for and sensitivity to rewarding experiences—is a core symptom of MDD. Notably, the presence of anhedonia has been shown as a marker of specificity distinguishing MDD from other psychiatric disorders [4], [5]. Further exploration of anhedonia is particularly important as anhedonic symptoms are less responsive to first-line antidepressants that act primarily on serotonergic or noradrenergic signaling pathways [6] and often persist after other depressive symptoms are in remission [7].

In recent years, investigators have focused on the objective characterization of anhedonic symptoms using quantitative behavioral and biological markers (e.g., Pizzagalli et al., 2005) [8]. Such studies have demonstrated that individuals with depressive symptoms exhibit diminished sensitivity to positive stimuli [9]–[15], impaired attentional bias towards positively valenced stimuli [16], and reduced behavioral and neurobiological responsiveness to probabilistic reward cues [8], [17]–[23].

These studies provide compelling empirical support for the notion that anhedonia is characterized by alterations in reward processing. However, the broad construct of “reward” is comprised of numerous distinct component processes, including reward learning, motivation, and hedonic response [24]. The studies cited above often utilized a measure of reward responsiveness as their primary dependent variable, and their findings have been interpreted as evidence that anhedonic symptoms are best construed as a blunting of the subjective hedonic response to reward. However, several studies that have directly assessed subjective pleasure responses in patient populations of individuals with MDD and matched controls have not found evidence to suggest that depression is associated with diminished hedonic capacity ([25], [26]). While further studies are required to clarify this issue, a critical concern for future research is the development of experimental designs that permit adequate dissociation of reward components.

Importantly, preclinical studies suggest that components of reward processing are mediated by dissociable neural systems, each of which may be differentially affected in the anhedonic endophenoptype. For example, while anhedonia is classically defined as reduced hedonic capacity (reward “liking”), it can also be viewed as decreased motivation to pursue rewards (reward “wanting”). The distinction between “liking” and “wanting” is strongly supported by animal models of reward processing, which have found that the dopaminergic (DA) system is critical for reward wanting, but is less involved in reward liking. In rodents, DA depletions leave hedonic responses to natural rewards intact, and do not reduce the readiness to consume easily available rewards [27], [28], [29]. In contrast, ventral striatal (nucleus accumbens) DA depletion results in a reduced willingness to expend effort in order to obtain rewards [30], [31], [32]. When given the option of performing little or no work for a small amount of reward or more work for a larger reward, animals with ventral striatal DA depletions consistently select the low effort option. This type of effort-based decision making represents a strong behavioral model of reduced “wanting” in animals.

Not surprisingly, several theorists have proposed that the symptoms of anhedonia in humans, specifically symptoms of reduced motivation or wanting, are related to a deficiency of DA signaling in the ventral striatum [33], [34]. However, direct clinical evidence for a DA hypothesis of anhedonia remains limited. The weak state of clinical evidence may arise for several reasons, including the frequent merging of wanting and liking deficits as a unitary construct, as is common to many self-report measures of positive affect and anhedonic symptoms (but see [35] for an important exception).

DA release in the nucleus accumbens (Nacc) has also been found to be sensitive to both the probability of reward receipt and the relative magnitude of the reward, such that the anticipation of relatively greater rewards under conditions of maximal uncertainty results in the greatest increase of sustained mesolimbic DA activity [36], [37]. Effort-based decision-making is similarly modulated by differences in reward magnitude [38] and relative risk [39]. If DA release is maximal during anticipation of high value, but highly unpredictable rewards, such a condition may be particularly sensitive to capturing individual differences in DA mediated reward circuitry. However, no previous research has specifically addressed whether probability of reward influences decision-making in relation to depression or anhedonia.

The present study has two primary aims: the first is to design an objective measure of effort-based decision-making that would specifically test the relationship between anhedonia and putative reward “wanting” in humans. The second was to demonstrate that the relationship between anhedonia and effort-based decision-making would be moderated by variables also known to influence Nacc DA release.

To achieve these goals, we developed the Effort-Expenditure for Rewards Task (EEfRT or “effort”). The EEfRT paradigm is based on a concurrent choice paradigm devised by Salamone and colleagues to explore effort-based decision-making in rodents [40]. In adapting this paradigm for use in humans, we presented subjects with a series of repeated trials in which they were able to choose between performing a “hard-task” or an “easy-task” in order to earn varying amounts of monetary rewards. In addition to varying reward magnitude, trials were presented with differing probability levels for reward receipt. This allowed us to examine the extent to which the relationship between anhedonia and effort-based decision-making was modulated by reward magnitude, probability of reward receipt and expected value.

Following this experimental design, we tested six Generalized Estimating Equation (GEE) models to explore the effects of these variables. The first model tested for main effects of probability, reward magnitude, expected value, and trait anhedonia as assessed by the Chapman Anhedonia scale [41] on the likelihood of choosing to expend greater effort for greater rewards. The second, third and fourth models tested for 2-way interactions between trait anhedonia and probability, reward magnitude, and expected value, respectively. Based on the results of these first four models, a fifth model tested for a 3-way interaction between trait anhedonia, probability and reward magnitude. Finally, in model six we performed an exploratory analysis of the relationship between the time-lagged effect of the prior trial and trait anhedonia.

## Methods

### Objectives and hypotheses

Based on the preclinical animal literature, we hypothesized that anhedonic traits would be associated with a reduced willingness to expend effort in order to obtain rewards. Specifically, when given a choice between expending little effort to obtain a small reward, or to expend greater effort to obtain a greater reward, individuals with higher levels of anhedonia should make fewer greater-effort/greater reward choices. We also hypothesized that the relationship between trait anhedonia and reduced effort expenditure would be modulated by probability and relative reward magnitude, and that this modulation would be strongest for trials that have high levels of reward uncertainty and high relative reward magnitude (and thus normally be associated with maximal DA firing), which would suggest a possible association between anhedonia and DA-mediated reward processes.

### Participants

61 participants (64% female) were recruited through Vanderbilt University and the community to participate in this study. Subjects were chosen from a larger sample of 324 undergraduates who were pre-screened using a brief self-report measure of hedonic responsiveness, the Snaith-Hamilton Pleasure Scale (SHAPS) [42]. This measure was used to ensure an appropriate range of trait anhedonia scores in our experimental sample.

### Ethics Statement

The Vanderbilt University Institutional Review Board approved the experimental protocol. A complete description of the study was provided to all participants, who all provided written informed consent.

### Self-report and Personality Measures

The Chapman physical and social anhedonia scales [41] served as the primary trait measure for anhedonia. We also included several other measures of anhedonia that are frequently used in the clinical literature, including the SHAPS, the Positive Affect Negative Affect Scale (PANAS scale;[43]), and the Beck Depression Inventory (BDI; [44]). In addition to the entire BDI, we investigated two subsets of items that have been associated with the Anhedonic endophenotype [8]. These included the BDI Anhedonia scale (items #4 – loss of pleasure, item #12 – loss of interest, item # 15 loss of energy and item #21 – loss of sex drive) and the BDI Melancholy scale (item (#4 – loss of pleasure, item #5 – presence of guilt, item # 11 – irritability, item #12 – loss of energy, item #16b – early waking and item #21 – loss of sex drive).

### Behavioral Measures: Effort-Expenditure for Rewards Task (“EEfRT”)

**Figure 1:**
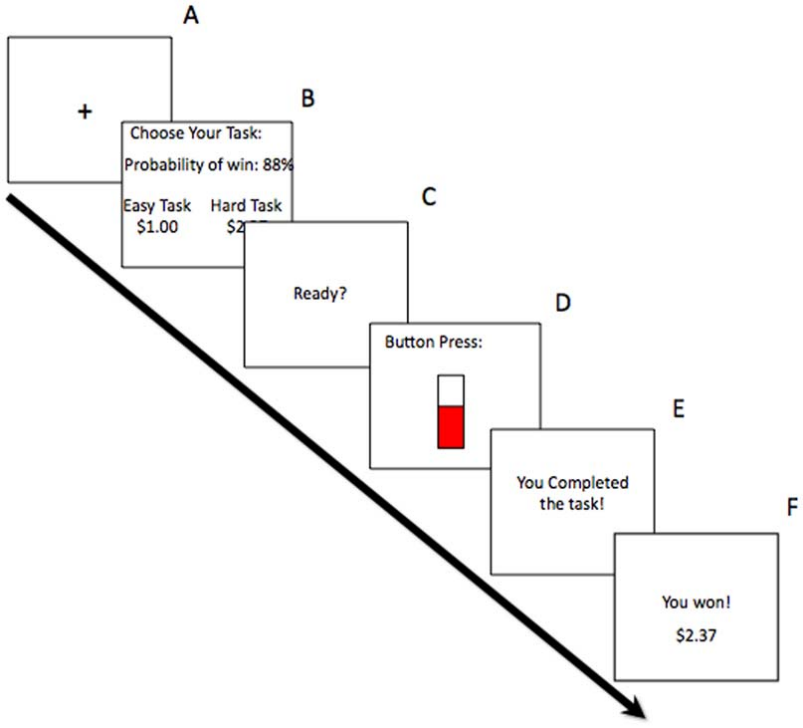
Schematic diagram of a single trial of the Effort Expenditure for Rewards Task (‘EEfRT’). A) Subjects begin by seeing a 1s fixation cue. B) 5s choice period in which subjects are presented with information regarding the reward magnitude of the hard task for that trial, and the probability of receiving any reward for that trial. C) 1s “ready” screen. D) Subjects make rapid button presses to complete the chosen task for 7s (easy task) or 21s (hard task). E) Subjects receive feedback on whether they have completed the task. F) Subjects receive reward feedback as to whether they received any money for that trial.

The EEfRT task is a multi-trial game in which participants are given an opportunity on each trial to choose between two different task difficulty levels in order to obtain monetary rewards (Figure 1). For all trials, participants made repeated manual button presses within a short period of time. Each button press raised the level of a virtual “bar” viewed onscreen by the participant. Participants were eligible to win the money allotted for each trial if they raised the bar to the “top” within the prescribed time period. Each trial presented the subject with a choice between two levels of task difficulty, a ‘hard task’ and an ‘easy task’. Successful completion of hard-task trials required the subject to make 100 button presses, using the non-dominant little finger within 21 seconds, while successful completion of easy-task trials required the subject to make 30 button presses, using the dominant index finger within 7 seconds.

For easy-task trials, subjects were eligible to win the same amount, $1.00, on each trial if they successfully completed the task. For hard-task choices, subjects were eligible to win higher amounts that varied per trial within a range of $1.24 – $4.30 (“reward magnitude”). Subjects were not guaranteed to win the reward if they complete the task; some trials were “win” trials, in which the subject received the stated reward amount, while others were “no win” trials, in which the subject received no money for that trial. To help subjects determine which trials were more likely to be win trials, subjects were provided with accurate probability cues at the beginning of each trial. Trials had three levels of probability: “high” 88% probability of being a win trial, “medium” 50% and “low” 12%. Probability levels always applied to both the hard task and easy task, and there were equal proportions of each probability level across the experiment. Each level of probability appeared once in conjunction with each level of reward value for the hard task. All subjects received trials presented in the same randomized order.

All trials began with a 1-second fixation cross, following a 5-second choice period in which subjects were presented with information regarding the probability of receiving reward and the reward magnitude of the hard task. Subjects were told that if they did not make a choice within 5 seconds, they would be randomly assigned to either the easy or the hard task for that trial. After making a choice, subjects were then shown a 1-second “Ready” screen and then completed the task. Following task completion, subjects were shown a 2 second feedback screen informing them that the task was successfully or unsuccessfully completed. If subjects successfully completed the task, then a second feedback screen appeared for 2 seconds in which subjects were told whether they had won money for that trial (reward feedback). In total, easy-task trials took approximately 15 seconds, whereas hard-task trials took approximately 30 seconds.

Subjects were told that they would receive a base-rate of compensation for their participation. In addition, they were told that two of their win trials would be randomly selected at the end of the experiment as “incentive trials,” for which they would receive the actual amount won on those trials. Subjects were informed that they had twenty minutes to play as many trials as they could. Since hard-task trials take approximately twice as much time to complete as easy-task trials, the number of trials that the subject was able to play depended in part on the choices that he or she made. This meant that making more hard-task trials toward the beginning of the experiment could reduce the total number of trials, which could in turn mean that the subject did not get a chance to play high-value, high-probability trials that might have appeared towards the end of the playing time. This trade-off was explained clearly to the subject. Importantly, subjects were not provided with any information regarding the distribution of trial types. The goal of this trade-off was to ensure that neither a strategy of always choosing the easy or the hard option could lead to an ‘optimal’ performance on the task. Moreover, the complexity of variables (with varying monetary reward levels, probability, and loss of time for future trials), does not lend itself to a formal calculation of an optimal response selection, and subjects were required to make decisions within a brief amount of time. This was done to help ensure that subject decisions reflected individual differences in the willingness to expend effort for a given level of expected reward value.

The EEfRT was programmed in Matlab (Matlab for Windows, Rel. 2007b. Mathworks Inc., Natick, MA) using the Psychtoolbox version 2.0.

### Study Procedure

Upon arriving to the lab, participants first reviewed a consent form and provided written consent. Participants were then asked to complete all self-report measures. After this, participants were provided with a series of task instructions. After participants read through the instructions, they were asked several simple questions to ensure they understood the task and its contingencies. Participants then played four practice trials. For the first two trials, the participant was instructed to choose the easy and hard task respectively, in order to gain familiarity with the level of effort required for each task. For the last two practice trials, the subject was free to choose. After completion of practice trials, the participant was asked if he or she had any questions. If not, then the subject commenced playing for a timed period of 20 minutes.

### Data Reduction and Analysis

Because subjects could only play for 20 minutes, the number of trials completed during that time varied from subject to subject (Mean trials completed  = 54, SD = 4.74, Range = 47–69 trials). For consistency of analysis, only the first 50 trials were used. Data were exported from Matlab into SPSS (SPSS for Macintosh, Rel. 16.0. 2008. Chicago: SPSS Inc.) for further analysis.

### Analysis Method 1: Repeated Measures ANOVA/Correlations

Data were analyzed using two statistical approaches. The first approach used repeated measures ANOVA and correlations. For these analyses, mean proportions of hard-task choices were created for all subjects across each level of probability. Proportions of hard-task choices and responses to self-report questionnaires were approximately normally distributed, and therefore parametric tests were used for inferential statistics.

### Analysis Method 2: Generalized Estimating Equations

The second approach used generalized estimating equations (GEE). GEE is a generalized regression model that is used to investigate continuous or logistic outcome variables in which the residuals are correlated [45], [46]. The term “Generalized” in this context means that different distributions (e.g. normal, dichotomous, Poisson) can be modeled through a link function. Importantly, GEE models allow for trial-by-trial modeling of both time-varying parameters (e.g., changes in reward value of the hard-task for each trial) as well as fixed effects (e.g., scores on anhedonia measures). GEE models were implemented in SPSS 16 using an unstructured working correlation matrix. The dependent measure was the dichotomous outcome of hard or easy task choice, and we used a binary logistic distribution to model the probability of choosing the hard-task. For all models, independent variables included probability, reward, expected value (reward magnitude X probability), trait anhedonia (Chapman) and gender. Separate models assessed the effects of trait anhedonia, and the interaction between trait anhedonia with probability level, reward magnitude and/or expected value. Additionally, we included an exploratory analysis that used a lagged independent variable coded for reward feedback on the previous trial, in order to determine if anhedonia interacted with prior reward history in influencing effort decisions.

### Effects of fatigue during the EEfRT

An important requirement for the EEfRT is that it measure individual differences in motivation for rewards, rather than individual differences in ability or fatigue. The task was specifically designed to require a meaningful difference in effort between hard and easy-task choices while still being simple enough to ensure that all subjects were capable of completing either task, and that subjects would not reach a point of exhaustion. Two manipulation checks were used to ensure that neither ability nor fatigue shaped our results. First, we examined the completion rate across all trials for each subject, and found that all subjects completed between 96%-100% of trials. This suggests that all subjects were readily able to complete both the hard and easy tasks throughout the experiment. As a second manipulation check, we used trial number as an additional covariate in each of our GEE models.

## Results

### Participants

Subject characteristics, and results of self-report measures appear in Table 1. Zero-order correlations between measures of mood and anhedonia are presented in Table 2. Due to experimenter error, BDI and SHAPS data were not available for three subjects.

**Table 1:** Demographic and Self Report Data.

Variable		*n*	*Mean*	*SD*
	Number of female participants	39 (64%)		
	Chapman Anhedonia Scales	61	19.5	11.6
	Beck Depression Inventory (BDI)	57	6.0	5.3
	BDI Anhedonia Subscale	57	1.2	1.3
	BDI Melancholy Subscale	57	1.3	1.4
	Snaith-Hamilton Pleasure Scale (SHAPS)	59	58.9	6.5
	PANAS Positive Affect	61	16.5	14.2
	PANAS Negative Affect	61	49.6	12.3

**Table 2:** Zero-order correlations between self-report measures.

Variable							
		BDI	BDI -An	BDI - Mel	SHAPS	PA	NA
	Chapman Anhedonia Scales	0.26*	0.29*	0.29*	−0.55^***^	0.15	0.25
	Beck Depression Inventory (BDI)		0.82^***^	0.82^***^	−0.38^**^	−0.28*	0.21
	BDI - Anhedonia Subscale			0.84^***^	−0.35*	−0.19	0.16
	BDI - Melancholy Subscale				−0.32*	−0.04	0.19
	SHAPS					0.23	−0.27*
	PANAS Postive Affect (PA)						−0.26*
	PANAS Negative Affect (NA)						

* *p*<.05, ^**^ *p*<.01, ^***^ *p*<.001.

### Main Effects of the EFFRT

A Repeated Measures ANOVA found a significant main effect for probability level on the proportion of hard task choices, with higher probability trials levels associated with more hard-task choices (*F*(2,120) = 139.8, *p*<.000, partial *η* ^2^ = 0.7). Across all subjects, proportion of hard-task choices for medium probability trials were moderately correlated with proportion of hard-task choices for both high probability (r = .31, *p*<.05) and low probability trials (*r* = .31, *p*<.05). High probability and low probability trials were uncorrelated (*r* = −.02, *p* = ns). We also found a main effect of gender, with men making more hard-task choices than women (*F*(1,59) = 3.9, *p* = .05). Consequently, gender was included as a covariate in all subsequent analyses.

### Effects of Trait Anhedonia

**Figure 2:**
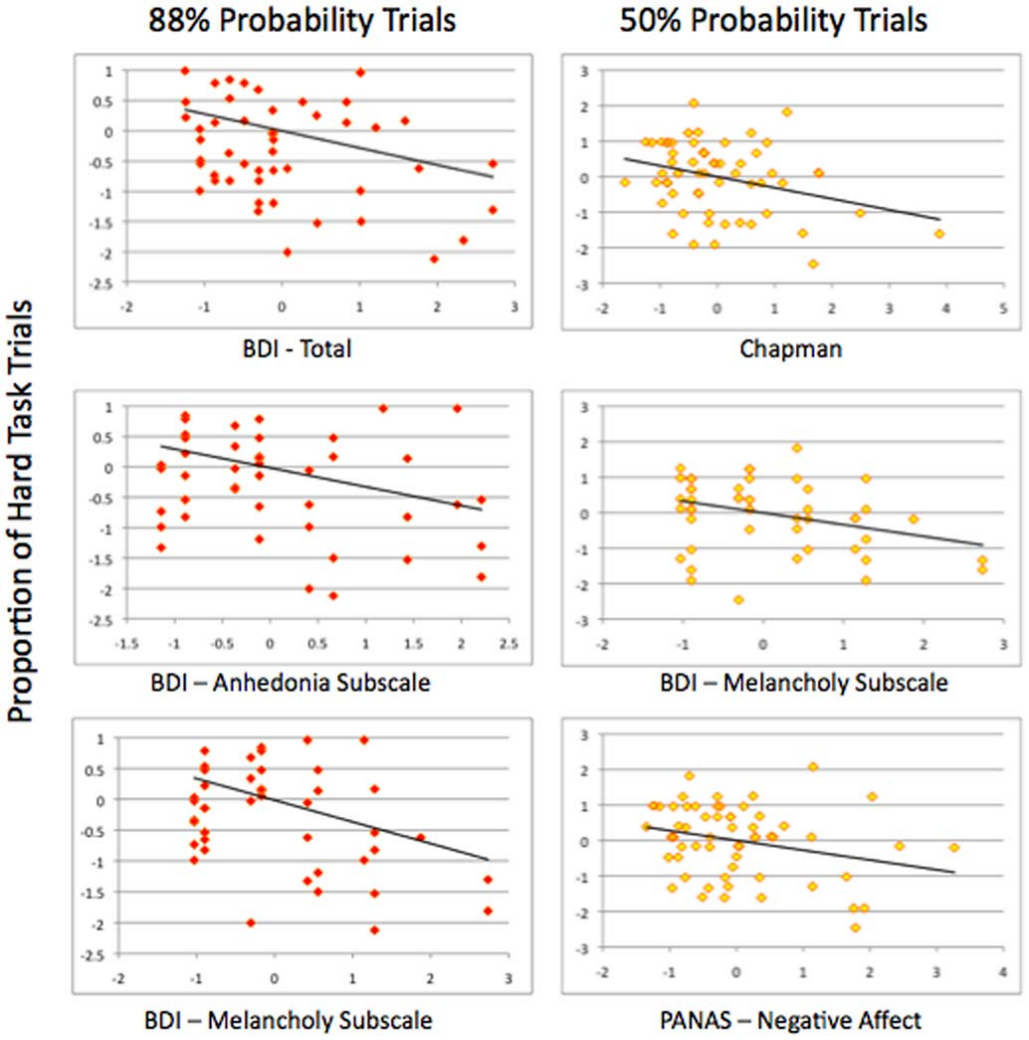
Partial regression plots between measures of anhedonia and proportion of hard-task choices, controlling for gender.

Partial correlations (controlling for gender) between proportion of hard task choices for each probability level and self-report measures of anhedonia, depression and positive affect appear in Table 3. The pattern of correlations appeared sensitive to the probability of winning for a given trial because the proportion of hard task choices was significantly inversely correlated with the BDI for high probability trials. For the medium probability trials, the proportion of hard-task choices correlated inversely with Chapman Anhedonia score, BDI melancholy items and reported negative affect. In contrast, there were no significant correlations for low probability trials. Scatter plots of significant correlations are presented in Figure 2.

**Table 3:** Correlations between self-report measures and proportion of hard-task choices.

Variable		Proportion of Hard Task Choices		
		88%	50%	12%
	Chapman Anhedonia Scales	−0.05	−.28*	−0.22
	Beck Depression Inventory (BDI)	−0.29*	−0.16	0.11
	BDI - Anhedonia Subscale	−0.31*	−0.22	0.09
	BDI - Melancholy Subscale	−.34*	−.34*	0.05
	SHAPS	0.16	0.13	−0.01
	PANAS Postive Affect (PA)	−0.08	−0.19	−0.22
	PANAS Negative Affect (NA)	0.03	−0.32*	−0.05

* *p*<.05. With *N* = 60, correlations as low as *r* = .36 have 80% power.

### Generalized Estimating Equations

We tested six separate models using generalized estimating equations (GEE). Each model included trial probability level, hard-task reward value and gender as covariates. Results of each model appear in Table 4.

**Table 4:** Generalized Estimating Equations.

		*b* Coefficient	SE	p
Model 1				
	Sex	0.323	0.09	0.001
	Trial Number	−0.006	0.00	0.006
	Probability	0.777	0.14	<0.001
	Reward	0.844	0.08	<0.001
	Expected Value	0.683	0.14	<0.001
	Chapman Anhedonia	−0.015	0.01	0.004
Model 2				
	Sex	0.298	0.01	0.001
	Trial Number	−0.005	0.00	0.009
	Probability	0.508	0.17	0.002
	Reward	0.857	0.08	<0.001
	Expected Value	0.686	0.14	<0.001
	Chapman Anhedonia	0.013	0.01	0.208
	Chapman Anhedonia ^*^ Probability	−0.014	0.01	0.005
Model 3				
	Sex	0.322	0.09	<0.001
	Trial Number	−0.007	0.00	0.002
	Probability	0.733	0.14	<0.001
	Reward	1.164	0.12	<0.001
	Expected Value	0.734	0.14	<0.001
	Chapman Anhedonia	0.031	0.01	0.017
	Chapman Anhedonia ^*^ Reward	−0.017	0.01	<0.001
Model 4				
	Sex	0.324	0.09	0.001
	Trial Number	−0.006	0.00	0.007
	Probability	0.778	0.14	<0.001
	Reward	0.846	0.08	<0.001
	Expected Value	0.646	0.16	<0.001
	Chapman Anhedonia	−0.017	0.01	0.046
	Chapman Anhedonia ^*^ Expected Value	0.002	0.01	0.702
Model 5				
	Sex	0.298	0.09	0.001
	Trial Number	−0.005	0.00	0.009
	Probability	0.754	0.14	<0.001
	Reward	1.144	0.11	<0.001
	Expected Value	0.467	0.15	0.001
	Chapman Anhedonia	0.011	0.01	0.123
	Chapman Anhedonia ^*^ Probability ^*^ Reward	−0.005	0.00	<0.001
Model 6				
	Sex	0.326	0.10	0.001
	Trial Number	−0.007	0.00	0.002
	Probability	0.790	0.14	<0.001
	Reward	0.859	0.08	<0.001
	Expected Value	0.686	0.13	<0.001
	Chapman Anhedonia	−0.015	0.01	0.004
	Prior Reward Feedback	−0.122	0.05	0.019
	Chapman Anhedonia ^*^ Prior Reward Feedback	0.012	0.00	<0.001

Model 1 tested for main effects of probability, reward magnitude, expected value (EV) and trait anhedonia. Increases in reward magnitude, probability of reward receipt and EV were significant predictors of making hard-task choices. We also found that increased trait anhedonia significantly predicted an overall reduced likelihood of making a hard-task choice (*b* = −.015, *p*<.005).

Model 2 tested for an interaction between trait anhedonia and probability level. The model revealed a significant anhedonia by probability interaction (b = −.014 p<.005). This interaction suggested that anhedonia significantly predicted trials at the 50% probability level (*b* = −.027 *p*<.01), and 12% level (*b* = −.035, *p*<.001) but not at the 88% level (*b* = −.008, *p* = ns).

Model 3 tested for an interaction between trait anhedonia and reward magnitude. A significant anhedonia by reward magnitude interaction (*b* = −.017, *p*<.001) emerged in this analysis, suggesting that anhedonia was a significant predictor of hard-task choices for trials in the upper half of reward values, (*b* = −.26, *p*<.000) but not in the lower half (*b* = 0.00, *p* = ns).

Model 4 tested for an interaction between trait anhedonia and EV. We did not find any evidence for an interaction between trait anhedonia and EV (*b* = .002, *p* = ns).

**Figure 3:**
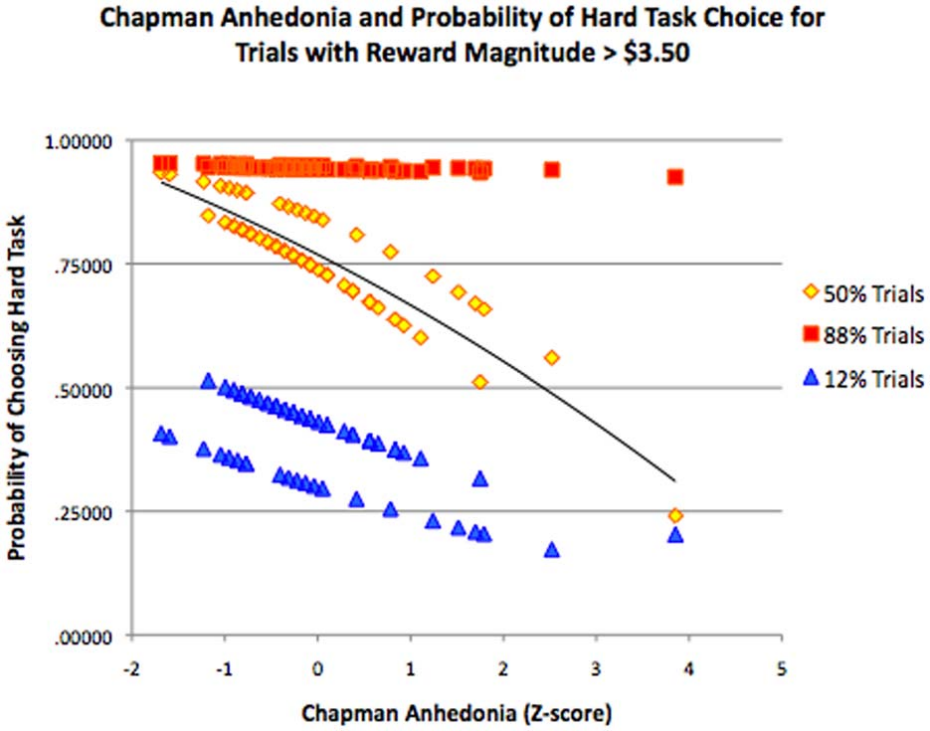
Relationship between Chapman anhedonia scores and GEE model predicted scores for trials with hard-task reward magnitudes >$3.50. Only trials at 50% probability level showed a significant relationship between anhedonia and model fit. The relationship between anhedonia and model fit for 50% probability trials was still significant after the outlier subject with the highest Chapman score was removed (*b* = −.052, *p* = .002). The presence of two lines both yellow and blue trials reflects differences in model fit due to gender.

Model 5 tested for a 3-way interaction between trait anhedonia, reward magnitude, and probability. This interaction was significant (*b* = −.005, *p*<.001). When restricting our analysis to examine only those trials for which the hard-task reward value was greater than $3.50, we found that trait anhedonia was a significant predictor for medium (50%) probability trials (*b* = −.054, *p* = .001), but not for high probability (b = −.006, *p* = ns) nor low probability trials (*b* = −.026, *p* = ns) (Figure 3).

Model 6 provided an exploratory analysis of the relationship between the time-lagged effect of the prior trial and trait anhedonia. We created a feedback regressor based on whether the subject received win or no-win feedback on the trial immediately preceding the current trial. The analysis revealed a significant interaction between win/no-win feedback and trait anhedonia (*b* = .01, *p*<.001), reflecting a greater influence of reward feedback on decision-making for individuals with higher levels of anhedonia. Using a median split based on the Chapman anhedonia scores, we divided our sample into two groups: low-anhedonia (LA) and high anhedonia (HA). We found that prior win/no-win feedback predicted hard task choices for the HA group (*b* = .127, *p*<.000), but not the LA group, (*b* = −.056 = 7, *p* = ns).

## Discussion

The present study had two specific aims: 1) to validate a novel effort-based decision-making task that could serve as an objective measure of individual differences in reward motivation; and 2) to explore interactions between anhedonia, probability and reward magnitude so as to determine whether these variables exhibited a pattern that would be consistent with preclinical models of Nacc DA release. In accordance with our first hypothesis, we found that individuals with elevated reports of both trait and state anhedonia exhibited a reduced willingness to make choices requiring greater effort in exchange for greater reward. This finding provides initial support for the EEfRT as a measure of putative reward “wanting”.

For the second aim, we explored the potential moderating effects of reward magnitude and probability, both of which have been shown to influence Nacc DA release during reward anticipation [47]. Preclinical models suggest that Nacc DA release is greatest for trials with high uncertainty and high reward magnitude [37]. Therefore, we hypothesized that the relationship between anhedonia and effort-based decision-making would be strongest for high reward trials at the 50% probability level. Consistent with this prediction we found significant two-way interactions between anhedonia and probability as well as anhedonia and reward magnitude. Further, we found a significant three-way interaction between anhedonia, probability and reward magnitude, such that anhedonia was the strongest predictor of hard-task choices for trials with maximal uncertainty (i.e. 50% probability) and maximum reward magnitude (hard task values >$3.50). It is also notable that the anhedonia coefficient for these high-reward, high-uncertainty trials (*b* = −.054) was much larger than the anhedonia coefficient for the experiment as a whole (*b* = −.015).

We did not find any evidence for an interaction between trait anhedonia and expected value. Prior neuroimaging studies in humans have suggested that BOLD signal in the ventral striatum is more sensitive to differing degrees of reward magnitude and probability, but not expected value, which is represented in regions of prefrontal and insular cortex [48], [49]. The specificity of the observed interactions between anhedonia and reward magnitude, probability, but not EV, is also consistent with the hypothesis that reduced reward motivation may be mediated in part by Nacc DA.

Although the present study did not directly assess DA functioning, significant prior evidence has linked the mesolimbic DA system to symptoms of anhedonia in depression [34], [50]–[52]. Additionally, our findings fit well with previous behavioral and neuroimaging studies that have reported associations between anhedonia and deficits in other DA-mediated processes, such as reward reinforcement learning [8], [23] and prediction error signals [20], [21]. Subsequent research will need to directly assess DA function in order to determine the role of DA as a potential mediator of performance on the EEfRT.

In an additional exploratory analysis, we found that the outcome of the previous trial significantly influenced willingness to make hard-task choices for individuals with higher levels of trait anhedonia, but not for individuals with lower levels. One interpretation is that individuals with higher levels of anhedonia have a heightened sensitivity to negative feedback from previous trials, and were thus less influenced by information about probability and reward magnitude when making decisions on subsequent trials. Such an explanation is consistent with the hypothesis that the anhedonic endophenotype is associated with impaired encoding of probabilistic reward cues [8], [17]–[23]. This result is also similar to studies suggesting that individuals with depression are more likely to commit errors on trials that follow negative feedback during memory, planning or reversal learning tasks [53]–[56]. In the context of the EEfRT, making an “error” following negative feedback (i.e., “no-win” feedback) might be viewed as a failure to appropriately suppress prior reward feedback when attempting to incorporate probability and reward value information presented on the current trial. Caution must be used in making this latter interpretation however, as the EEfRT has only “win” and “no-win” trials, and therefore we cannot interpret the association between prior trial feedback and hard-task choices in individuals with higher levels of anhedonia as a reflection of sensitivity for exclusively negative outcomes.

We also found a main effect of gender across all analyses, with women consistently making fewer hard-task choices than men. Given that the EEfRT is a computer-based task that emphasizes physical performance, it is conceivable that the task is gender-biased. Additional studies will determine whether these observed differences stem from particular design elements of the EEfRT, or reflect a true gender disparity in normative effort-based decision-making.

### Limitations

The present study has several limitations. First, our participants were recruited from a non-clinical sample, with a lower range of scores on anhedonia measures than would be expected in individuals with MDD. Additional research will be required to demonstrate the utility of the EEfRT in characterizing the anhedonic endophenotype within clinical populations. A second limitation is the relative complexity of the EEfRT task in comparison with the tasks used by Salamone and colleagues. We felt this complexity was necessary to prevent the use of optimization strategies. However, it is still conceivable that some subjects attempted to determine an optimal strategy, which may reduce the specificity of the EEfRT as a behavioral measure of anhedonia. Finally, we note that our primary measure in this initial validation study was a self-report measure of anhedonia. While the observed association between trait anhedonia and performance on the EEfRT provides evidence for the construct validity of the paradigm, it will eventually need to be shown that the task not only correlates with anhedonic symptoms, but demonstrates incremental validity and utility over and beyond existing self-report measures.

### Conclusions

The present study unveiled a novel effort-based decision-making task, the ‘EEfRT’, as a means of exploring effort-based decision-making in humans. Based on a well-validated animal paradigm, the EEfRT operationalized reduced reward ‘wanting’ as a decreased willingness to choose greater-effort/greater-reward options, particularly when rewards are uncertain. Consistent with our hypotheses, we found that individuals with self-reported anhedonia made fewer hard-task choices. These findings are consistent with theoretical models linking anhedonia to decreased mesolimbic DA function. As an objective measure of individual differences in reward motivation, we believe the EEfRT may provide a useful tool for studying DA functioning and motivation, as well characterizing the endophenotype of anhedonia, and its responsiveness to clinical treatment.
